# Some Results of Nurses' Insurance

**Published:** 1921-06-18

**Authors:** 


### Some Results of Nurses' Insurance.

We call to mind that the nursing world received with extreme disfavour the news that nurses were to be included in the provisions of the National Health Insurance Act, and a good many attempts were made to escape from its jurisdiction. It is all the more interesting to record the exceptional success which was brought into view at the ninth annual meeting of the Nurses' Insurance Society.

The membership of the Society now exceeds 40,000. The increase of the normal rate of benefit from 7s. 6d. to 12s. per week and the disablement from 5s. to 7s. 6d. a week has brought about an increase of some £4,000 in benefits paid out. The sickness rate has by no means increased in proportion to the increase in membership. Owing to this fact and the circumstance presumably that nurses understand better than others how to keep themselves in good health, Sir Thomas Dewey, the Chairman, was able to report a surplus of £32,779.

Members will, we believe, learn with real satisfaction that three-sevenths of this sum, about £14,000, is to be allocated to supplying such as need this assistance with free dental treatment and dentures. The necessity for such treatment comes as a heavy blow to all whose income is limited, and nurses often for this reason defer attending to their teeth too long and suffer greatly in consequence. Two-sevenths of the surplus, or about £9,000, is to be used for procuring hospital benefits for private nurses and others to whom this aid is not secured under agreement in institutions. The remaining two-sevenths will be used for the purpose of making allowances during convalescence, the need for which has been abundantly illustrated.

Thus it appears that the once despised grant during sickness is but one of the valuable forms of protection afforded to nurses by their insurance society.

